# Quantitative Neutron Dark-field Imaging through Spin-Echo Interferometry

**DOI:** 10.1038/srep16576

**Published:** 2015-11-12

**Authors:** Markus Strobl, Morten Sales, Jeroen Plomp, Wim G. Bouwman, Anton S. Tremsin, Anders Kaestner, Catherine Pappas, Klaus Habicht

**Affiliations:** 1 European Spallation Source AB, Lund 22100, Sweden; 2 University of Copenhagen, The Niels Bohr Institute, Copenhagen 2100, Denmark; 3 Helmholtz Centre Berlin for Materials and Energy (HZB), Berlin 14109, Germany; 4 Delft University of Technology, Reactor Institut Delft, Delft 2629, The Netherlands; 5 University of California at Berkeley, Space Sciences Laboratory, Berkeley 94720, California, USA; 6 Paul Scherrer Institut, Villigen-PSI 5232, Switzerland

## Abstract

Neutron dark-field imaging constitutes a seminal progress in the field of neutron imaging as it combines real space resolution capability with information provided by one of the most significant neutron scattering techniques, namely small angle scattering. The success of structural characterizations bridging the gap between macroscopic and microscopic features has been enabled by the introduction of grating interferometers so far. The induced interference pattern, a spatial beam modulation, allows for mapping of small-angle scattering signals and hence addressing microstructures beyond direct spatial resolution of the imaging system with high efficiency. However, to date the quantification in the small angle scattering regime is severely limited by the monochromatic approach. To overcome such drawback we here introduce an alternative and more flexible method of interferometric beam modulation utilizing a spin-echo technique. This novel method facilitates straightforward quantitative dark-field neutron imaging, i.e. the required quantitative microstructural characterization combined with real space image resolution. For the first time quantitative microstructural reciprocal space information from small angle neutron scattering becomes available together with macroscopic image information creating the potential to quantify several orders of magnitude in structure sizes simultaneously.

The promise of neutron dark-field imaging^1^ is to provide access to structural features from the macroscopic range probed with image resolution to the microscopic range in the micrometer and sub-micrometer range and hence beyond direct spatial resolution of the order of 10 micrometers^2^. This can be provided through the registration of scatter signals in the (ultra)-small angle neutron scattering ((U-)SANS) regime which allows to close the gap between real space and reciprocal space methods. The corresponding size range is of significant importance to hierarchical structures and when probing real systems, be it in soft matter, biology or engineering and non-destructive testing of devices. Accordingly neutron dark-field imaging with grating interferometers^1^, the first corresponding method offering an efficiency suitable for the relatively low phase space density of neutron sources, has experienced a remarkable impact, especially in engineering^3^ and magnetic structure characterizations^4^,^5^, strongholds of neutron applications. The fact that such wealth of results could be obtained up to now with only qualitative information in the scattering regime underlines the outstanding potential as well as the explicit need for quantitative solutions^6^. Therefore we are introducing a novel interferometer method for imaging based on neutron spin-echo principles, that is analogue to the grating interferometry approach, but, due to its higher flexibility, can provide full quantitative SANS characterization in the dark-field regime and is well suited for time-of-flight (ToF) measurements. ToF is a neutron technique, which requires a pulsed beam and allows for intrinsic neutron energy resolution and hence for coverage of a significant scattering range simultaneously. Therefore our method will be able to take advantage of the most powerful new generation of pulsed neutron sources. However, the present proof-of-principle has been undertaken at the low-flux thermal neutron source of the Reactor Institute of Delft Technical University (RID/TUD) and could be quantified successfully despite of the low available brightness, which in turn is proving the efficiency of the method.

The principle of interferometric dark-field imaging with a spatially modulated beam is, that scattering to small angles will spatially redistribute intensities between the maxima and minima and hence dampens the amplitude of the beam modulation. Attenuation and differential phase signals can be recorded simultaneously but are well separated from the dark-field effect as these generally decrease the mean intensity or shift the modulation phase, respectively. Hence a systematic study of the effects on the spatial modulation allows for extracting attenuation, differential phase and the dark-field signal as a measure of small angle scattering separately. While Talbot Lau grating interferometers induce a cosine spatial beam modulation through phase and absorption gratings^7^, in Spin-Echo-Modulation SANS (SEMSANS) such modulation is introduced by interference conditions induced by spin precession devices for a polarized neutron beam^8^,^9^. It has been demonstrated earlier that such approach can provide quantitative SANS characterizations in analogy to the well-established Spin-Echo SANS (SESANS) technique^10^ even in a highly efficient ToF mode^9^,^11^, and that in analogy to grating interferometry also a grating analyser can be utilized when required in order to resolve the beam modulation^12^. It has in turn been demonstrated that in principle corresponding quantification is equally possible with grating interferometric dark-field imaging^6^. However gratings must be optimized for a single wavelength^7^, a condition, which seems to severely limit straightforward broad application. For the work presented here a ToF SEMSANS instrument was modified in order to enable simultaneous real space image resolution. In contrast to earlier set-ups the sample position has been moved to behind the spin analyser and hence closer to the detector. Together with the beam collimation defined by a narrow entrance slit into the spin manipulation devices and a ToF imaging detector^13^ this set-up allowed to achieve the additionally required spatial resolution. However, the new sample position also enables investigations of magnetic samples, which might depolarize the beam, as beam polarization is not relevant anymore at that location of the sample.

The basic set-up depicted schematically in Fig. 1 consists of a polarizer and polarization analyzer, between which the spin polarized neutrons pass through two key precession fields which are directed in opposite directions. These precession field regions have a triangular shape, which guarantees, that (i) neutrons arriving at the same point in the detector have experienced the same total spin rotation and that (ii) this final rotation angle is to a first approximation a linear and continuous function of the lateral position across the beam on the detector. Hence the polarization analyzer located downstream of the second precession field induces a one-dimensional cosine modulation of the beam on the detector. To achieve such regular modulation the focusing condition L_1_B_1_ = L_2_B_2_, where L_1_ and L_2_ and B_1_ and B_2_ are the distances to the detector and the field values of the two precession devices, respectively, has to be satisfied. The period ζ of the modulation can then be shown to be^8^,^9^


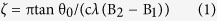


with θ_0_ the inclination of the precession field surfaces to the beam, λ the wavelength and 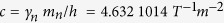 with *γ*_*n*_ and *m*_*n*_ the gyromagnetic ratio and the mass of the neutron and 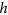 the Planck constant. Together with the sample to detector distance L_s_ a spin-echo length δ^SE^ in analogy to SESANS^10^ can be defined to be


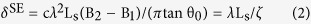


and denotes the correlation length probed with the corresponding settings. This establishes not only a complete analogy with SESANS on the one hand but also to grating interferometry with respect to the corresponding autocorrelation length that can be defined accordingly^6^. Both parameters, the period and the spin echo length are wavelength dependent with a linear and square dependence, respectively. Consequently, in a ToF configuration, intrinsically varying the wavelength over a certain range, allows to probe corresponding ranges of correlation lengths, i.e. structure sizes in the sample in the SANS regime. On the other hand also the magnetic fields and distances can be used to tune correlation lengths correspondingly and to tailor the range to the experimental requirements. This way the method provides an extensive flexibility which is required to probe a large range of length scales and which is lacking in the case of grating interferometers.

## Measurements

For this work a ToF approach was chosen utilizing optical blind pulse shaping choppers providing a wavelength resolution for the given instrument length of about Δλ/λ≈5%^9^. The thermal source spectrum provided sufficient neutron flux for data recorded for wavelengths from 1.6 Å to 5.25 Å, and hence spin-echo lengths δ^SE^, i.e. a structure size range, from about 15 nm to 180 nm could be probed. This corresponds to distances and magnetic fields set to L_1_ = 5 m, L_2_ = 1.7 m, L_s_ = 0.55 m and B_1_ = 1.43 mT, B_2_ = 4.22 mT. In contrast to conventional grating interferometry operating in the micrometer range, the corresponding modulation periods from 5.7 mm to 1.6 mm could be resolved directly by the spatial resolution of the detector^13^. However, for smaller modulation periods and longer spin-echo lengths achieved e.g. with higher magnetic fields or longer wavelengths, also grating analysers like in the Talbot Lau interferometer case can be used. This has been demonstrated earlier for SEMSANS measurements^12^. For the presented case data analysis and image formation require intense post processing, as the real space image is superposed by the modulation. On the other hand no scanning approach like in the case of the utilization of grating analyzers is required.

It can be shown that the relative modulation visibility with respect to the empty beam measurement V_s_/V_0_, with the visibility being V=(I_max_ − I_min_)/(I_max_ + I_min_), can be written, again in analogy to the grating interferometer case^6^ and SESANS^8^,^10^ as


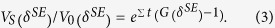


Here, Σ and t represent the scattering power (total scattering cross section) and the sample thickness, respectively, while G is the real space correlation function of the scattering structures or particles^6^,^9^. Hence, the normalized visibility V_s_/V_0_ is depending on the spin-echo length and thus is a direct measure of the scattering power Σ and real space correlation function G representing the scattering structures. Therefore analyzing the visibility locally in the recorded images allows full structural characterization in the SANS range probed in real space for any lateral location in the sample.

**Figure 2:**
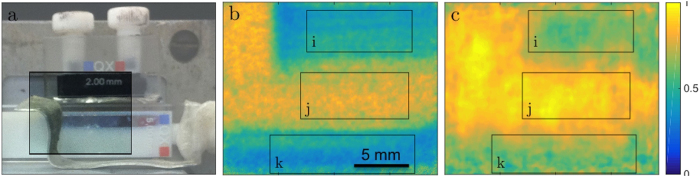
Sample images. (**a**) Sample set-up photo with the exposed area highlighted. (**b**) Attenuation contrast image of exposed region. (**c**) Dark-field SEMSANS image displaying the visibility of the spin-echo modulation at a certain spin-echo length. Three areas of interest are highlighted in (**b, c**): powder sample (i), empty beam area (j) and PS dispersion (k).

**Figure 3:**
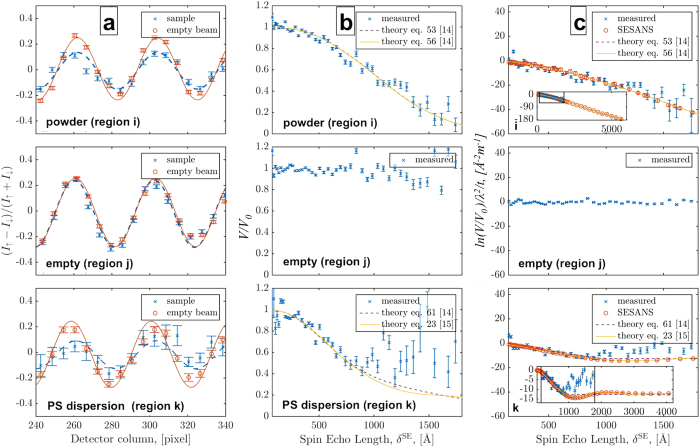
Data and results. The rows from top to bottom correspond to the powder sample, open beam area and PVC dilution and hence to (parts) of the areas marked in Fig. 2 as i, j and k respectively. (**a**) Examples of local fits of normalized data at 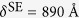. (**b**) Extracted local visibility behavior and corresponding model fits. (**c**) Corrected local SEMSANS curves compared to SESANS measurements which provide the correct sample parameters in terms of scattering cross section Σ and real space correlation function.

For the measurements we used a sample consisting of two quartz cuvettes on top of each other (Fig. 2). The top cuvette contained a magnetic metal powder (Ferroxdure YXF1, BaFe_12_O_19_, Yuxiang Magnetic Materials Ind. CO Ltd) with a probed thickness of 2 mm and an average grain size of the order of micrometers according to the producer. The lower cuvette was lying on its side and contained a solution of spherical polystyrene (PS-DVB) particles with defined monodispersive diameters of 136 nm in D_2_O with a weight concentration of 12.4% and a sample thickness of 5 mm. Note that the metal pieces (Cd) visible in the photograph in Fig. 2a have been removed after alignment of the sample which they aided. Additionally, the liquid sample displayed a significant meniscus between the walls of the cuvette, with a correspondingly reduced effective sample thickness in these areas, which is clearly represented in the attenuation contrast image (Fig. 2b), but also in the dark-field SEMSANS image (Fig. 2c). Measurements were performed for spin-up 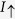 and spin-down 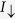 neutrons, i.e. two opposite incoming spin polarizations, which corresponds to a modulation phase shift of π between the two measurements. Hence, using the sum of the two measurements the attenuation contrast image 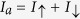 can be extracted straightforwardly (Fig. 2b). Despite the overlaying beam modulation with wavelength dependent periods, and integrating over all time bins, i.e. the full utilized neutron spectrum, an image that can be referred to as a “white beam” radiography is provided. In addition the two opposite images in terms of the modulation can be used to calculate an attenuation corrected modulation signal 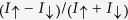 (Fig. 3a). Open beam visibilities V_0_, mainly limited by the polarization quality, but also by experimental conditions like e.g. the detector resolution, have been found to be between 65% at 1.6 Å and 20% at 5.25 Å in the measurements. Measurement times for each of the two empty beam and sample measurements were of the order of 3.5 hours and can be performed much faster e.g. at state-of-the-art spallation neutron sources with several orders of magnitude higher pulsed brightness.

## Results

The resulting image data sets were analyzed in terms of the local modulation, in particular their local visibility. For this purpose a cosine fitting routine has been developed, which is capable of shifting the area of interest, typically the width of one period for the shortest utilized wavelength (i.e. longest spatial period for a specific measurement) pixel by pixel over the full image. This way the modulation parameters can be extracted pixel by pixel as a running average over the given width, which in turn limits the spatial resolution achievable with such approach. However, due to the image blur introduced by the geometry of the setup, especially by the relatively long sample to detector distance with respect to a conventional imaging experiment, the spatial resolution was in any case limited to about 1 mm. In contrast to that the high pixel resolution of the detector of 55 micrometer was required to resolve the beam modulation. The results of corresponding fitting routines are displayed in the first column of Fig. 3, while the second column displays the extracted relative visibility as a function of the spin-echo length probed by the ToF approach for several individual but representative image locations. To achieve quantitative results the latter have to be corrected by the sample thickness and wavelength dependence of the total scattering, which leads to the results in column 3 of Fig. 3 for three areas of interest in the sample, namely the powder sample (top), a sample-free area (middle) and the PVC dispersion (bottom). These final curves show good agreement with the complimentary SESANS measurements and theory curves describing the structural features of 1 μm and 136 nm for the metallic powder and the PS dispersion, respectively. The theoretical curves were derived with respect to the best fit using models for highly concentrated spheres (eq. 61 in Ref. 14)taking into account next neighbor correlations in the case of the dilution of 12.4% PS in D_2_O (eq. (23) in Ref. 15) and for simple random two-phase media (eq. (53) in Ref. 14) as well as random two-phase media with Hurst exponent based on eq. (56) in ref. 14 for the powder sample. These are conventional models used to interpret small-angle scattering data, but adapted to Spin-Echo SANS. Applying these models to the data (Fig. 3b,c) suggest structure sizes of around 1 μm, as implied by the manufacturer, for the powder and between 110 nm and 160 nm for the PS particles with nominal diameters of 136 nm. The total scattering values deduced are in good agreement with the 12.4 wt.% concentration of the particles.

## Conclusion

From these seminal results which unambiguously prove the principle and potential of the method, we conclude that the presented method not only unlocks the access to an intermediate size range for structural investigations, but also bridges an unprecedented size range between the macroscopic and microscopic scales, which is invaluable for the investigation of real systems and components as in contrast to homogeneous model samples of materials required for corresponding microscopic investigations otherwise. These proof-of-principle measurements with low flux at a thermal source and the limited capacity of the prototype magnetic set-up allow to extrapolate to an optimized set-up in the sense that at a powerful state-of-the-art pulsed source of cold neutrons the gap between real space and Fourier space investigations through scattering can be closed with a single set-up and even tomographic 3D structural investigations in this range might prove feasible. A correspondingly available flux increase of about 3 to 4 orders of magnitude in particular for longer wavelengths and magnetic fields tunable to values of about one order of magnitude higher, together will allow not only for improved statistics, shorter exposure times and for extending the covered size range of the SE analyses from the nanometer into the micrometer range, but also for extending the real space resolution from the millimeter to the micrometer range, like it is state of the art at dedicated instruments. This will pave the way for unprecedented observations of microstructural evolutions and inhomogeneities. The ToF approach, covering orders of magnitude in size simultaneously also enables kinetic studies of structural developments as neutrons enable a wide range of sample environments, applying pressures, temperatures, fields, strains, and shear like required in many fields of engineering, soft matter and magnetism investigations. The evolution of magnetic domains, crystallographic grains, pores and voids as well as precipitates or coagulation and clustering on the corresponding microscopic length scales can be studied with simultaneous macroscopic resolution of process inhomogeneities. Kinetics and structure evolutions can be separated into local ones versus global in a sample, depending on external parameters and inherent structural features, and hence also studies of complex assemblies like biological or engineering systems and components become feasible in this range.

## Additional Information

**How to cite this article**: Strobl, M. *et al.* Quantitative Neutron Dark-field Imaging through Spin-Echo Interferometry. *Sci. Rep.* **5**, 16576; doi: 10.1038/srep16576 (2015).

## Figures and Tables

**Figure 1:**
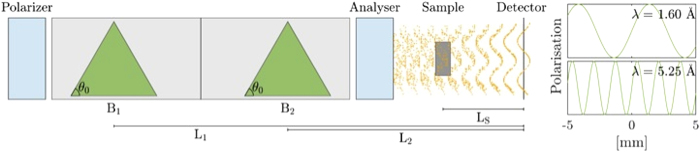
Schematic illustration of experimental set-up for SEMSANS-imaging including the polarizer and analyzer as well as triangularly shaped magnetic field regions and the induced spatial beam modulation in the area of the sample and detector. On the right hand side an example of beam modulations is given for two particular wavelengths.
